# Supplementary material for: Nutritional Values of Minikiwi Fruit (Actinidia arguta) after Storage: Comparison between DCA New Technology and ULO and CA
Source: Molecules. 2022 Jul 5;27(13):4313. doi: 10.3390/molecules27134313 (PMC9268200; doi:10.3390/molecules27134313)
Supplement: Supplementary file 1 [file molecules-27-04313-s001.zip › molecules-1773983-supplementary.pdf]

Table S1. Changes in antioxidants activity (mg vit C·100 g^{-1} F.W.), measured in 'Geneva' and 'Ananasnaya' minikiwi fruit.

Cultivars	Storage Conditions	Period of Storage (Weeks)					
		0	4	6	8	10	12
		**2017**					
Geneva	DCA		0.69 ± 0.11	0.76 ± 0.02	0.71 ± 0.06	0.63 ± 0.06	0.64 ± 0.06
	ULO	0.85 ± 0.05	0.76 ± 0.03	0.71 ± 0.02	0.69 ± 0.15	0.68 ± 0.02	0.55 ± 0.04
	CA		0.79 ± 0.10	0.64 ± 0.04	0.75 ± 0.04	0.75 ± 0.04	0.78 ± 0.02
	2018						
	DCA		1.01 ± 0.14	1.04 ± 0.07	1.06 ± 0.08	0.89 ± 0.07	0.90 ± 0.07
	ULO	1.15 ± 0.12	1.06 ± 0.05	1.08 ± 0.04	1.04 ± 0.19	0.98 ± 0.05	0.80 ± 0.05
	CA		1.18 ± 0.15	0.96 ± 0.07	1.13 ± 0.12	1.07 ± 0.05	1.11 ± 0.07
Ananasnaya	2017						
	DCA		0.76 ± 0.09	0.75 ± 0.03	0.71 ± 0.03	0.69 ± 0.03	0.63 ± 0.04
	ULO	0.80 ± 0.06	0.73 ± 0.07	0.62 ± 0.02	0.69 ± 0.15	0.60 ± 0.07	0.62 ± 0.04
	CA		0.85 ± 0.04	0.77 ± 0.02	0.83 ± 0.06	0.81 ± 0.02	0.83 ± 0.06
	2018						
	DCA		0.82 ± 0.09	0.82 ± 0.01	0.81 ± 0.03	0.82 ± 0.02	0.75 ± 0.01
	ULO	0.94 ± 0.07	0.80 ± 0.06	0.78 ± 0.04	0.76 ± 0.11	0.63 ± 0.02	0.71 ± 0.03
	CA		0.94 ± 0.05	0.87 ± 0.04	0.92 ± 0.08	0.92 ± 0.03	0.95 ± 0.04

DCA, dynamic controlled atmosphere, 0.4% CO_2:0.4% O_2; ULO, ultra-low oxygen, 1.5% CO_2:1.5% O_2; CA, controlled atmosphere, 5% CO_2:1.5% O_2; data are presented as mean ± standard deviation.

Table S2. Changes in ascorbic acid (mg ·100 g^{-1} F.W.), measured in 'Geneva' and 'Ananasnaya' minikiwi fruit.

Cultivars	Storage Conditions	Period of Storage (Weeks) 0	4	6	8	10	12
		**2017**					
Geneva	DCA		57.1 ± 6.61	59.4 ± 0.51	58.6 ± 1.46	53.3 ± 0.72	50.6 ± 0.72
	ULO	67.8 ± 6.12	62.6 ± 1.68	54.9 ± 1.56	54.1 ± 9.20	50.5 ± 0.79	47.5 ± 0.79
	CA		67.2 ± 5.62	59.9 ± 3.37	66.5 ± 8.65	63.0 ± 0.33	61.8 ± 0.36
		2018					
	DCA		64.6 ± 7.51	67.1 ± 0.57	66.3 ± 1.61	60.4 ± 0.87	57.3 ± 0.83
	ULO	70.1 ± 2.95	70.9 ± 1.87	62.1 ± 1.79	61.2 ± 10.4	57.2 ± 0.95	53.7 ± 0.90
	CA		76.0 ± 6.37	67.9 ± 3.80	75.3 ± 9.77	71.3 ± 0.38	69.9 ± 0.41
		2017					
Ananasnaya	DCA		73.2 ± 5.73	70.6 ± 3.78	73.5 ± 2.56	65.7 ± 0.96	62.1 ± 0.96
	ULO	85.3 ± 8.06	70.0 ± 4.22	65.9 ± 1.59	66.3 ± 11.0	58.9 ±1.24	54.3 ± 1.24
	CA		84.4 ± 4.53	81.2 ± 5.61	84.4 ± 14.1	82.8 ± 0.17	82.2 ± 0.15
		2018					
	DCA		59.9 ± 9.61	61.6 ± 0.97	59.1 ± 2.92	60.0 ± 2.52	57.8 ± 3.02
	ULO	70.2 ± 8.30	60.3 ± 4.09	56.1 ± 5.33	58.4 ± 3.73	47.5 ± 5.30	51.2 ± 4.48
	CA		75.2 ± 3.92	65.4 ± 6.47	70.3 ± 11.2	69.4 ± 1.13	72.6 ± 3.37

DCA, dynamic controlled atmosphere, 0.4% CO_2:0.4% O_2; ULO, ultra-low oxygen, 1.5% CO_2:1.5% O_2; CA, controlled atmosphere, 5% CO_2:1.5% O_2; data are presented as mean ± standard deviation.

Table S3. Changes in TPC (mg ·100 g^{-1} F.W.), measured in 'Geneva' and 'Ananasnaya' minikiwi fruit.

Cultivars	Storage Conditions	Period of Storage (Weeks) 0	4	6	8	10	12
		**2017**					
Geneva	DCA		93.1 ± 1.67	85.3 ± 2.42	88.4 ± 9.46	87.4 ± 1.05	84.4 ± 1.35
	ULO	110.1 ± 7.42	92.1 ± 2.25	86.6 ± 5.91	94.7 ± 12.2	78.1 ± 1.48	72.2 ± 1.11
	CA		110.5 ± 6.85	109.0 ± 7.11	109.8 ± 16.8	110.3 ± 0.19	109.3 ± 0.56
		2018					
	DCA		107.3 ± 1.91	98.2 ± 2.73	101.8 ± 10.8	97.1 ± 1.36	92.5 ± 1.65
	ULO	120.1 ± 0.99	106.1 ± 2.77	99.5 ± 6.77	110.3 ± 15.73	93.5 ± 1.45	87.9 ± 1.14
	CA		127.3 ± 7.94	125.7 ± 8.16	126.5 ± 19.3	126.9 ± 0.24	125.9 ± 0.50
		2017					
Ananasnaya	DCA		82.3 ± 6.45	76.3 ± 2.97	78.31 ± 14.95	80.9 ± 6.22	75.5 ± 2.19
	ULO	101.9 ± 7.90	85.2 ± 2.10	82.5 ± 8.42	95.8 ± 19.1	70.7 ± 1.12	65.9 ± 0.99
	CA		105.9 ± 5.41	102.2 ± 7.29	105.7 ± 17.6	90.6 ± 0.31	90.2 ± 0.05
		2018					
	DCA		88.1 ± 5.50	82.1 ± 3.8	82.6 ± 16.1	80.3 ± 4.18	75.0 ± 0.66
	ULO	106.1 ± 7.98	90.8 ± 1.16	87.9 ± 8.70	101.9 ± 18.4	85.8 ± 2.40	78.3 ± 1.16
	CA		109.2 ± 9.37	109.5 ± 6.84	114.5 ± 17.9	102.4 ± 2.64	103.5 ± 2.74

DCA, dynamic controlled atmosphere, 0.4% CO_2:0.4% O_2; ULO, ultra-low oxygen, 1.5% CO_2:1.5% O_2; CA, controlled atmosphere, 5% CO_2:1.5% O_2; data are presented as mean ± standard deviation; TPC, total phenolic content.

Table S4. Changes in phenolic acids (mg ·100 g^{-1} F.W.), measured in 'Geneva' and 'Ananasnaya' minikiwi fruit.

Cultivars	Storage Conditions	Period of Storage (Weeks) 0	4	6	8	10	12
		**2017**					
Geneva	DCA		2.02 ± 0.11	1.78 ± 0.09	1.89 ± 0.12	1.90 ± 0.05	1.70 ± 0.01
	ULO	2.32 ± 0.14	2.11 ± 0.12	1.90 ± 0.15	1.94 ± 0.22	1.78 ± 0.09	1.63 ± 0.06
	CA		2.28 ± 0.13	2.41 ± 0.13	2.33 ± 0.20	2.38 ± 0.08	2.30 ± 0.05
		2018					
	DCA		3.25 ± 0.17	2.68 ± 0.13	2.82 ± 0.30	2.59 ± 0.14	2.58 ± 0.08
	ULO	3.38 ± 0.21	3.34 ± 0.37	2.92 ± 0.33	3.11 ± 0.49	3.02 ± 0.12	2.65 ± 0.13
	CA		3.53 ± 0.34	3.70 ± 0.22	3.62 ± 0.38	3.78 ± 0.03	3.59 ± 0.15
Ananasnaya		2017					
	DCA		1.60 ± 0.12	1.29 ± 0.08	1.42 ± 0.13	1.42 ± 0.09	1.31 ± 0.03
	ULO	1.76 ± 0.05	1.71 ± 0.12	1.60 ± 0.10	1.65 ± 0.24	1.39 ± 0.08	1.21 ± 0.00
	CA		1.92 ± 0.16	1.89 ± 0.12	2.02 ± 0.25	1.90 ± 0.02	1.79 ± 0.04
		2018					
	DCA		1.75 ± 0.20	1.35 ± 0.04	1.46 ± 0.27	1.48 ± 0.30	1.33 ± 0.30
	ULO	1.59 ± 0.17	1.68 ± 0.27	1.57 ± 0.15	1.75 ± 0.08	1.67 ± 0.15	1.35 ± 0.12
	CA		1.74 ± 0.34	1.81 ± 0.15	2.10 ± 0.07	1.85 ± 0.23	2.00 ± 0.15

DCA, dynamic controlled atmosphere, 0.4% CO_2:0.4% O_2; ULO, ultra-low oxygen, 1.5% CO_2:1.5% O_2; CA, controlled atmosphere, 5% CO_2:1.5% O_2; data are presented as mean ± standard deviation.

Table S5. Changes in flavonols (mg ·100 g⁻¹ F.W.), measured in 'Geneva' and 'Ananasnaya' minikiwi fruit.

Cultivars	Storage Conditions	Period of Storage (Weeks) 0	4	6	8	10	12
		**2017**					
Geneva	DCA		1.63 ± 0.11	1.41 ± 0.07	1.51 ± 0.14	1.46 ± 0.10	1.49 ± 0.03
	ULO	1.66 ± 0.06	1.56 ± 0.15	1.42 ± 0.03	1.58 ± 0.16	1.46 ± 0.05	1.37 ± 0.13
	CA		1.75 ± 0.03	1.85 ± 0.05	1.81 ± 0.22	1.75 ± 0.08	1.76 ± 0.08
		2018					
	DCA		1.21 ± 0.08	1.10 ± 0.04	1.15 ± 0.08	1.09 ± 0.04	1.08 ± 0.02
	ULO	1.26 ± 0.03	1.20 ± 0.08	1.08 ± 0.02	1.18 ± 0.14	1.09 ± 0.03	1.03 ± 0.06
	CA		1.34 ± 0.05	1.35 ± 0.05	1.36 ± 0.17	1.32 ± 0.04	1.31 ± 0.03
		2017					
Ananasnaya	DCA		5.36 ± 0.47	4.88 ± 0.21	5.20 ± 0.77	4.85 ± 0.22	4.39 ± 0.01
	ULO	6.58 ± 0.55	5.82 ± 0.09	5.27 ± 0.43	6.20 ± 0.92	5.28 ± 0.09	5.20 ± 0.05
	CA		6.51 ± 0.40	6.27 ± 0.52	6.67 ± 1.08	6.40 ± 0.07	6.22 ± 0.18
		2018					
	DCA		5.32 ± 0.35	4.59 ± 0.25	5.20 ± 0.97	4.64 ± 0.28	4.32 ± 0.03
	ULO	6.73 ± 0.74	5.44 ± 0.11	5.10 ± 0.66	6.17 ± 0.94	5.29 ± 0.29	5.48 ± 0.16
	CA		6.37 ± 0.52	6.19 ± 0.72	6.49 ± 0.98	6.32 ± 0.12	6.18 ± 0.23

DCA, dynamic controlled atmosphere, 0.4% CO_2:0.4% O_2; ULO, ultra-low oxygen, 1.5% CO_2:1.5% O_2; CA, controlled atmosphere, 5% CO_2:1.5% O_2; data are presented as mean ± standard deviation.

Table S6. Changes in flavan-3-ols (mg ·100 g^{-1} F.W.), measured in 'Geneva' and 'Ananasnaya' minikiwi fruit.

Cultivars	Storage Conditions	Period of Storage (Weeks) 0	4	6	8	10	12
		**2017**					
Geneva	DCA		0.42 ± 0.01	0.38 ± 0.03	0.42 ± 0.04	0.39 ± 0.01	0.38 ± 0.02
	ULO	0.50 ± 0.01	0.42 ± 0.03	0.39 ± 0.06	0.42 ± 0.05	0.38 ± 0.02	0.36 ± 0.02
	CA		0.47 ± 0.03	0.49 ± 0.05	0.47 ± 0.06	0.47 ± 0.03	0.49 ± 0.02
		2018					
	DCA		0.42 ± 0.02	0.39 ± 0.02	0.41 ± 0.05	0.38 ± 0.01	0.34 ± 0.03
	ULO	0.50 ± 0.01	0.41 ± 0.03	0.39 ± 0.05	0.43 ± 0.06	0.39± 0.01	0.37 ± 0.01
	CA		0.46 ± 0.03	0.50 ± 0.03	0.48 ± 0.06	0.50 ± 0.01	0.49 ± 0.00
		2017					
Ananasnaya	DCA		0.47 ± 0.02	0.43 ± 0.02	0.42 ± 0.06	0.43 ± 0.02	0.40 ± 0.03
	ULO	0.54 ± 0.03	0.48 ± 0.03	0.46 ± 0.05	0.52 ± 0.08	0.40 ± 0.01	0.39± 0.02
	CA		0.55 ± 0.03	0.54 ± 0.03	0.57 ± 0.08	0.49 ± 0.00	0.51 ± 0.01
		2018					
	DCA		0.56 ± 0.01	0.49 ± 0.04	0.52 ± 0.09	0.46 ± 0.02	0.40 ± 0.02
	ULO	0.62 ± 0.06	0.53 ± 0.05	0.50 ± 0.07	0.57 ± 0.12	0.49 ± 0.02	0.47 ± 0.03
	CA		0.61 ± 0.03	0.62 ± 0.01	0.66 ± 0.01	0.60 ± 0.03	0.62 ± 0.01

DCA, dynamic controlled atmosphere, 0.4% CO_2:0.4% O_2; ULO, ultra-low oxygen, 1.5% CO_2:1.5% O_2; CA, controlled atmosphere, 5% CO_2:1.5% O_2; data are presented as mean ± standard deviation.

Table S7. Changes in glucose (g ·100 g^{-1} F.W.), measured in 'Geneva' and 'Ananasnaya' minikiwi fruit.

Cultivars	Storage Conditions	Period of Storage (Weeks) 0	4	6	8	10	12
		**2017**					
Geneva	DCA		2.66 ± 0.10	3.04 ± 0.02	3.63 ± 0.00	3.47 ± 0.01	3.25 ± 0.03
	ULO	1.91 ± 0.04	2.71 ± 0.19	3.11 ± 0.06	3.69 ± 0.05	3.74 ± 0.12	3.89 ± 0.05
	CA		2.66 ± 0.09	2.92 ± 0.06	3.39 ± 0.03	3.55 ± 0.02	3.61 ± 0.06
		2018					
	DCA		2.55 ± 0.45	2.90 ± 0.08	3.55 ± 0.02	3.46 ± 0.10	3.55 ± 0.04
	ULO	2.02 ± 0.08	2.86 ± 0.16	3.28 ± 0.04	3.74 ± 0.02	3.61 ± 0.02	3.55 ± 0.05
	CA		2.50 ± 0.10	2.75 ± 0.12	3.36 ± 0.08	3.46 ± 0.05	3.67 ± 0.07
		2017					
Ananasnaya	DCA		2.04 ± 0.04	2.43 ± 0.02	2.83 ± 0.04	2.75 ± 0.03	2.75 ± 0.01
	ULO	1.55 ± 0.04	2.35 ± 0.08	2.67 ± 0.04	2.74 ± 0.04	2.69 ± 0.03	2.49 ± 0.02
	CA		2.11 ± 0.05	2.37 ± 0.03	2.85 ± 0.05	3.01 ± 0.01	3.08 ± 0.01
		2018					
	DCA		1.86 ± 0.08	2.26 ± 0.02	2.72 ± 0.02	2.69 ± 0.02	2.83 ± 0.05
	ULO	1.52 ± 0.03	2.12 ± 0.10	2.44 ± 0.06	2.84 ± 0.03	2.85 ± 0.04	2.91 ± 0.06
	CA		1.94 ± 0.03	2.16 ± 0.04	2.78 ± 0.06	2.83 ± 0.05	3.03 ± 0.06

DCA, dynamic controlled atmosphere, 0.4% CO_2:0.4% O_2; ULO, ultra-low oxygen, 1.5% CO_2:1.5% O_2; CA, controlled atmosphere, 5% CO_2:1.5% O_2; data are presented as mean ± standard deviation.

Table S8. Changes in fructose (g ·100 g^{-1} F.W.), measured in 'Geneva' and 'Ananasnaya' minikiwi fruit.

Cultivars	Storage Conditions	Period of Storage (Weeks)					
		0	4	6	8	10	12
		**2017**					
Geneva	DCA	2.30 ± 0.03	2.93 ± 0.15	3.12 ± 0.09	3.56 ± 0.07	3.59 ± 0.05	3.62 ± 0.06
	ULO		3.00 ± 0.07	3.39 ± 0.09	3.94 ± 0.09	3.95 ± 0.07	4.17 ± 0.06
	CA		2.87 ± 0.02	3.07 ± 0.02	3.51 ± 0.03	3.58 ± 0.04	3.70 ± 0.07
		2018					
	DCA	2.53 ± 0.04	3.21 ± 0.14	3.47 ± 0.09	4.03 ± 0.06	4.03 ± 0.07	4.09 ± 0.07
	ULO		3.32 ± 0.10	3.76 ± 0.09	4.40 ± 0.08	4.42 ± 0.09	4.65 ± 0.05
	CA		2.15 ± 0.03	3.39 ± 0.05	3.93 ± 0.03	4.02 ± 0.04	4.18 ± 0.07
Ananasnaya		2017					
	DCA	2.20 ± 0.07	2.89 ± 0.05	3.17 ± 0.05	3.63 ± 0.07	3.50 ± 0.06	3.43 ± 0.03
	ULO		3.17 ± 0.10	3.53 ± 0.03	3.53 ± 0.04	3.47 ± 0.03	3.17 ± 0.02
	CA		2.88 ± 0.05	3.13 ± 0.07	3.66 ± 0.06	3.94 ± 0.05	4.13 ± 0.06
		2018					
	DCA	1.98 ± 0.05	2.49 ± 0.07	2.72 ± 0.06	3.17 ± 0.10	3.16 ± 0.03	3.22 ± 0.04
	ULO		2.66 ± 0.06	2.98 ± 0.05	3.36 ± 0.05	3.31 ± 0.05	3.33 ± 0.06
	CA		2.46 ± 0.07	2.66 ± 0.08	3.22 ± 0.10	3.34 ± 0.05	3.55 ± 0.05

DCA, dynamic controlled atmosphere, 0.4% CO_2:0.4% O_2; ULO, ultra-low oxygen, 1.5% CO_2:1.5% O_2; CA, controlled atmosphere, 5% CO_2:1.5% O_2; data are presented as mean ± standard deviation.

Table S9. Changes in sucrose (g ·100 g^{-1} F.W.), measured in 'Geneva' and 'Ananasnaya' minikiwi fruit.

Cultivars	Storage Conditions	Period of Storage (Weeks) 0	4	6	8	10	12
		**2017**					
Geneva	DCA		7.02 ± 0.36	6.50 ± 0.20	7.48 ± 0.18	7.17 ± 0.24	7.16 ± 0.35
	ULO	8.45 ± 0.31	6.52 ± 0.29	5.53 ± 0.28	6.98 ± 0.40	6.46 ± 0.28	5.58 ± 0.34
	CA		7.50 ± 0.50	6.77 ± 0.05	7.86 ± 0.12	7.79 ± 0.22	7.39 ± 0.32
		2018					
	DCA		6.93 ± 0.40	6.69 ± 0.29	6.72 ± 0.17	6.46 ± 0.15	6.27 ± 0.23
	ULO	8.00 ± 0.32	6.72 ± 0.15	6.14 ± 0.21	6.37 ± 0.21	5.90 ± 0.09	5.55 ± 0.15
	CA		7.34 ± 0.34	6.79 ± 0.09	7.04 ± 0.15	6.86 ± 0.22	6.52 ± 0.25
		2017					
Ananasnaya	DCA		4.93 ± 0.33	3.96 ± 0.20	3.93 ± 0.32	3.99 ± 0.10	4.05 ± 0.33
	ULO	6.67 ± 0.03	4.11 ± 0.23	3.60 ± 0.38	3.50 ± 0.30	3.25 ± 0.13	2.70 ± 0.30
	CA		5.45 ± 0.30	4.46 ± 0.16	5.42 ± 0.40	4.86 ± 0.05	4.44 ± 0.33
		2018					
	DCA		5.57 ± 0.18	4.89 ± 0.10	4.70 ± 0.19	4.46 ± 0.09	4.37 ± 0.20
	ULO	6.64 ± 0.04	5.00 ± 0.13	4.24 ± 0.09	4.29 ± 0.11	4.05 ± 0.11	3.57 ± 0.18
	CA		5.95 ± 0.20	5.24 ± 0.18	5.55 ± 0.25	5.21 ± 0.05	4.94 ± 0.16

DCA, dynamic controlled atmosphere, 0.4% CO_2:0.4% O_2; ULO, ultra-low oxygen, 1.5% CO_2:1.5% O_2; CA, controlled atmosphere, 5% CO_2:1.5% O_2; data are presented as mean ± standard deviation.

Table S10. Changes in citric acid (g ·100 g^{-1} F.W.), measured in 'Geneva' and 'Ananasnaya' minikiwi fruit.

Cultivars	Storage Conditions	Period of Storage (Weeks)					
		0	4	6	8	10	12
		2017					
Geneva	DCA		0.950 ± 0.087	0.793 ± 0.085	0.746 ± 0.047	0.744 ± 0.020	0.625 ± 0.058
	ULO	1.145 ± 0.09	0.819 ± 0.093	0.794 ± 0.082	0.770 ± 0.052	0.589 ± 0.071	0.628 ± 0.086
	CA		0.885 ± 0.048	0.786 ± 0.104	0.855 ± 0.020	0.849 ± 0.037	0.727 ± 0.057
		2018					
	DCA		0.731 ± 0.016	0.695 ± 0.014	0.659 ± 0.016	0.606 ± 0.004	0.525 ± 0.021
	ULO	0.871 ± 0.01	0.694 ± 0.019	0.679 ± 0.030	0.611± 0.021	0.579 ± 0.031	0.520 ± 0.020
	CA		0.790 ± 0.027	0.716 ± 0.055	0.687 ± 0.015	0.661 ± 0.023	0.648 ± 0.017
		2017					
Ananasnaya	DCA		0.668 ± 0.004	0.572 ± 0.017	0.551 ± 0.030	0.542 ± 0.041	0.486 ± 0.015
	ULO	0.739 ± 0.02	0.590 ± 0.046	0.556 ± 0.018	0.503 ± 0.015	0.443 ± 0.007	0.334 ± 0.035
	CA		0.676 ± 0.028	0.659 ± 0.044	0.613 ± 0.035	0.580 ± 0.053	0.550 ± 0.008
		2018					
	DCA		0.734 ± 0.012	0.672 ± 0.026	0.692 ± 0.059	0.637 ± 0.072	0.537 ± 0.024
	ULO	0.884 ± 0.06	0.713 ± 0.017	0.677 ± 0.049	0.568 ± 0.039	0.476 ± 0.004	0.421 ± 0.066
	CA		0.781 ± 0.046	0.794 ± 0.074	0.717 ± 0.050	0.729 ± 0.053	0.688 ± 0.009

DCA, dynamic controlled atmosphere, 0.4% CO_2:0.4% O_2; ULO, ultra-low oxygen, 1.5% CO_2:1.5% O_2; CA, controlled atmosphere, 5% CO_2:1.5% O_2; data are presented as mean ± standard deviation.

Table S11. Changes in malic acid (g ·100 g^{-1} F.W.), measured in 'Geneva' and 'Ananasnaya' minikiwi fruit.

Cultivars	Storage Conditions	Period of Storage (Weeks) 0	4	6	8	10	12
		**2017**					
Geneva	DCA		0.124 ± 0.018	0.113 ± 0.004	0.108 ± 0.011	0.093 ± 0.004	0.083 ± 0.012
	ULO	0.150 ± 0.01	0.108 ± 0.003	0.113 ± 0.018	0.108 ± 0.002	0.088 ± 0.011	0.081 ± 0.007
	CA		0.127 ± 0.005	0.130 ± 0.019	0.118 ± 0.006	0.122 ± 0.008	0.107 ± 0.010
		2018					
	DCA		0.117 ± 0.010	0.115 ± 0.002	0.109 ± 0.006	0.097 ± 0.016	0.077± 0.007
	ULO	0.131 ± 0.01	0.103 ± 0.008	0.103 ± 0.005	0.102 ± 0.006	0.082 ± 0.014	0.059 ± 0.005
	CA		0.120 ± 0.013	0.124 ± 0.006	0.110 ± 0.009	0.109 ± 0.014	0.104 ± 0.013
Ananasnaya		2017					
	DCA		0.188 ± 0.023	0.180 ± 0.018	0.140 ± 0.013	0.169 ± 0.017	0.140 ± 0.009
	ULO	0.220 ± 0.020	0.188 ± 0.017	0.150 ± 0.013	0.137 ± 0.028	0.134 ± 0.016	0.105 ± 0.009
	CA		0.238 ± 0.012	0.260 ± 0.005	0.174 ± 0.006	0.201 ± 0.018	0.183 ± 0.027
		2018					
	DCA		0.117 ± 0.003	0.104 ± 0.006	0.083 ± 0.003	0.081 ± 0.008	0.075 ± 0.007
	ULO	0.127 ± 0.01	0.103 ± 0.008	0.085 ± 0.006	0.072 ± 0.004	0.060 ± 0.003	0.056 ± 0.009
	CA		0.125 ± 0.006	0.132 ± 0.006	0.097 ± 0.004	0.097 ± 0.006	0.096 ± 0.008

DCA, dynamic controlled atmosphere, 0.4% CO_2:0.4% O_2; ULO, ultra-low oxygen, 1.5% CO_2:1.5% O_2; CA, controlled atmosphere, 5% CO_2:1.5% O_2; data are presented as mean ± standard deviation.
